# *Lactobacillus* fermentation enhances the inhibitory effect of Hwangryun-haedok-tang in an ovariectomy-induced bone loss

**DOI:** 10.1186/1472-6882-13-106

**Published:** 2013-05-16

**Authors:** Ki-Shuk Shim, Taesoo Kim, Hyunil Ha, Kwang Jin Lee, Chang-Won Cho, Han Sung Kim, Dong-Hyun Seo, Jin Yeul Ma

**Affiliations:** 1 KM-Based Herbal Drug Research Group, Korea Institute of Oriental Medicine, Daejeon 305-811, South Korea; 2 Regional Food Industry Research Group, Korea Food Research Institute, Sungnam 463-746, South Korea; 3 Department of Biomedical Engineering, Institute of Medical Engineering, Yonsei University, Wonju, Gangwon 220-710, South Korea; 4 Yonsei-Fraunhofer Medical Device Lab, Yonsei University, Wonju Gangwon, 220-710, South Korea

**Keywords:** Hwangryun-haedok-tang, *Lactobacillus curvatus*, Osteoclastogenesis, RANKL, Ovariectomy

## Abstract

**Background:**

Hwangryun-haedok-tang (HRT) is traditional herbal medicine used to treat inflammatory-related diseases in Asia. However, its effect on osteoclastogenesis and bone loss is still unknown. In this study, we evaluated the effect of HRT and its fermented product (fHRT) on the receptor activator for the nuclear factor-κB ligand-induced osteoclastogenesis using murine bone marrow-derived macrophages and postmenopausal bone loss using an ovariectomy (OVX) rat model.

**Methods:**

Tartrate resistant acid phosphatase (TRAP) staining was employed to evaluate osteoclast formation. mRNA level of transcription factor and protein levels of signaling molecules were determined by real-time quantitative polymerase chain reaction and Western blot analysis, respectively. Effect of HRT or fHRT on OVX-induced bone loss was evaluated using OVX rats orally administered HRT, or fHRT with 300 mg/kg for 12 weeks. Micro-CT analysis of femora was performed to analyze bone parameter.

**Results:**

HRT or fHRT treatment significantly decreased TRAP activity and the number of TRAP positive multinuclear cells on osteoclastogenesis. Interestingly, these inhibitory effects of HRT were enhanced by fermentation. Furthermore, fHRT significantly inhibited mRNA and protein expression of nuclear factor of activated T cells cytoplasmic 1, which leads to down-regulation of NFATc1-regulated mRNA expressions such as TRAP, the d2 isoform of vacuolar ATPase V(0) domain, and cathepsin K. Administration of fHRT significantly inhibited the decrease of bone mineral density, and improved bone parameter of femora more than that of HRT and vehicle in OVX rats.

**Conclusions:**

This study demonstrated that lactic bacterial fermentation fortifies the inhibitory effect of HRT on osteoclastogenesis and bone loss. These results suggest that fermented HRT might have the beneficial potential on osteoporosis by inhibiting osteoclastogenesis.

## Background

Osteoporosis is a bone disease characterized by an impaired bone strength and reduced bone mass that cause skeletal fragility and increase the risk of fracture [1]. Estrogen deficiency underlying etiology of postmenopausal osteoporosis increases bone turnover with relative increase of osteoclastic bone resorption than osteoblastic bone formation. It results in an imbalance of bone remodeling, leading to a net loss of bone mineral density (BMD) and disruption of bone micro-structure [2]. Estrogen replacement therapy (ERT) is widely used to prevent and to treat postmenopausal osteoporosis [3]. However, due to several adverse effects of ERT, the Women's Health Initiative recommends that the benefits and risks of ERT should be carefully weighed and non-hormonal treatment is considered as an alternative way for managing postmenopausal osteoporosis [4]. There are growing interests in the traditional medicine as alternative treatment for osteoporosis [5]. Traditional herbal medicine has an inhibitory effect on osteoclastic bone resorption and/or estrogen deficiency-induced bone loss, which suggests the beneficial potential of traditional medicine on postmenopausal osteoporosis [6].

Osteoclasts differentiate from the monocyte/macrophage lineage of hematopoietic stem cells in the presence of receptor activator for nuclear factor-κB ligand (RANKL) and macrophage colony-stimulating factor (M-CSF) [7]. RANK/RANKL signaling plays a major role in osteoclastogenesis through the activation of downstream signaling cascades, including mitogen-activated protein (MAP) kinases (ERK [extracellular signal-regulated kinase], JNK [c-Jun N-terminal kinase], and p38) and NF-κB [8]. RANKL/RANK signaling pathway consequently stimulates key transcription factors, activation protein-1 (AP-1) and nuclear factor of activated T cells (NFATc1) [7,8]. AP-1 positively regulates osteoclastogenesis by transcriptionally inducing NFATc1 expression during early stage of osteoclastogenesis. NFATc1 is a master transcription factor that regulates a series of osteoclastogenesis-specific genes, including tartrate-resistant acid phosphatase (TRAP), the d2 isoform of vacuolar ATPase V(0) domain (ATP6v0d2), and cathepsin K. NFATc1 rescues osteoclastogenesis in cells lacking c-Fos, indicating that c-Fos is upstream of NFATc1 [9].

Hwangryun-haedok-tang (HRT) is a traditional herbal medicine used to treat inflammatory-related diseases, including dermatitis, hepatitis, and gastritis. Investigation on pharmacological effect of HRT suggests that HRT inhibits an inflammatory response by decreasing eicosanoid and nitric oxide production in carrageenan-injected rat model [10]. However, there is no study that evaluates whether HRT affects on RANKL-induced osteoclastogenesis.

Fermentation has been suggested as a traditional process for enhancing the pharmacological effect of herbal medicine based on the theory of the Oriental medicine [11]. Fermentation increases the production of active components in medicinal herbs, which beneficially influences health through health-promoting and disease preventing effects. A body of evidence enhancing pharmacological effect of medicinal herbs by fermentation includes an antioxidant effect of *Anoectochilus formosanus* Hayata fermented by *Lactobacillus acidophilus*[12], an anabolic effect of antler fermented by *Cordyceps militaris* on osteoblast differentiation [13], and anti-aging effect of Radix astragali fermented by *Bacillus subtilis* natto in human skin cells [14]. However, there is no investigation on whether bacterial fermentation could change the pharmacological effect of traditional herbal medicine on osteoclastogenesis and ovariectomy (OVX)-induced bone loss.

This study investigated the inhibitory effect of HRT on RANKL-induced osteoclastogenesis and whether the bacterial fermentation could affect this effect of HRT. In a preliminary study, we found that *Lactobacillus curvatus* fermented HRT (fHRT) exerts an enhancement of inhibitory effect compared to non-fermented HRT. Furthermore, we selected *n*-butanol as the organic solvent for maximizing the inhibitory effect of fHRT on osteoclastogenesis. In this study, we explored the inhibitory effect of *n*-butanol extract of HRT (HRT-BU) and fHRT (fHRT-BU) on mRNA level of NFATc1, c-Fos, and osteoclastogenesis-related genes, the activation of MAP kinases and NF-κB to establish the underlying molecular mechanism of fHRT-BU using mouse bone marrow-derived macrophages (BMMs). To evaluate the therapeutic potential of HRT and fHRT for treating postmenopausal osteoporosis, we examined the inhibitory effect of HRT and fHRT on OVX-induced bone loss.

## Methods

### Reagents

*Coptis japonica Makino*, *Phellodendron chinense Schneider*, *Gardenia jasminoides fructus*, and *Scutellaria baicalensis Georgi* were purchased from Dongkyung Inc. (Seoul, Korea). General aerobic MRS medium and MRS agar were purchased from Difco Co. (Detroit, MI, USA). Geniposide, baicalin, palmatine, berberine, wogonoside, baicalein, and wogonin were purchased from Tianjin Pharma Tech. Co. (China). 1,25(OH)_2_D_3_, polybrene, puromycin, *p*-nitrophenyl phosphate, and TFA were purchased from Sigma Chemical Co. (St. Louis, MO, USA). All antibodies were purchased from Cell Signaling Technology (Danvers, MA, USA) except NFATc1 (7A6) and c-Fos (H-125) antibodies purchased from Santa-cruz Biotechnology Inc. (CA, USA).

### Preparation of HRT

*Coptis japonica Makino 2*50 g, *Scutellaria baicalensis Georgi* 250 g, *Phellodendron chinense Schneider* 250 g, and *Gardenia jasminoides fructus* 250 g were used in this study. All voucher specimens were deposited in the herbal bank of KM-Based Herbal Drug Research Group, Korea Institute of Oriental Medicine (No. 2010–144, 166, 168, and 170). HRT was prepared by using a water extraction method (Gyeongseo Extractor Cosmos-600, Inchon, Korea). The total quantity of medicinal herbs was placed in 10 L of distilled water for 1 h, and then extracted by heating for 3 h at 115°C. After extraction, HRT was filtered out using standard testing sieves (106 μm) (Retsch, Haan, Germany) and stored at 4°C before use (yield about 19.42%).

### Fermentation of HRT

*Lactobacillus curvatus* KFRI-166 (Korea Food Research Institute, Seongnam-si, Korea) was used for fermentation of HRT. After two successive transfers of the test organisms in MRS broth at 37°C for 24 h, the activated cultures were again inoculated into broth. It was properly diluted to obtain an initial population of 1–5 × 10^6^ CFU/ml and served as the inoculum. Viable cell counts of the strains were determined in duplicate by using the pour plate method on MRS agar. For fermentation, 5 ml of HRT was inoculated with 0.05 ml of the inoculums that was incubated at 37°C for 48 h. HRT or fHRT was fractionated by successive solvent extraction with *n* -hexane, ethylacetate, and *n*-butanol. Each fraction evaporated to dryness under vacuo was stored in desiccators at −20°C before use. The *n*-butanol soluble fraction of HRT and fHRT was designated as HRT-BU (yield about 16.45%) and fHRT-BU (yield about 17.23%), respectively.

### HPLC analysis

The Waters HPLC system (Waters Co., USA) consisting of a pump, autosampler, column oven, and diode array UV/VIS detector. The output signal of the detector was recorded using Empower software from Waters. Chromatographic separation was achieved in a Luna C_18_ column (4.6 mm × 250 mm, 5 μm, Phenomenex Co., USA) at 254 nm. The mobile phase was 100% acetonitrile (A) and water containing 0.1% trifluoroacetic acid (B) with a step gradient elution (0–10 min, 90% B; 10–25 min, 80% B; 25-33 min, 70% B; 33-43 min, 67% B; 43–50 min, 60% B; and 50–60 min, 56% B) at a flow rate of 1.0 ml/min. Each standard solution was prepared by dissolving each marker components, geniposide (1), baicalin (2), palmatine (3), berberine (4), wogonoside (5), baicalein (6) and wogonin (7), with 100% methanol at the concentration of 0.2 mg/ml. Prior to analysis, the sample was filtered through a 0.2 μm PVDF filter and 10 μL of samples was injected for the HPLC analysis. We prepared two batches of HRT, fHRT, HRT-BU, and fHRT-BU under the same condition and analyzed marker components in each batch by HPLC analysis. We successfully detected geniposide (1, *t*_R_ 17.51 min), baicalin (2, *t*_R_ 36.10 min), palmatine (3, *t*_R_ 39.59 min), berberine (4, *t*_R_ 39.77 min), wogonoside (5, *t*_R_ 41.19 min), baicalein (6, *t*_R_ 48.44 min), and wogonin (7, *t*_R_ 58.5 min) in HRT and fHRT. In addition, we also detected geniposide (*t*_R_ 17.44 min), baicalin (*t*_R_ 36.05 min), palmatine (*t*_R_ 39.95 min), berberine (*t*_R_ 40.35 min), and wogonoside (*t*_R_ 41.11 min) in HRT-BU and fHRT-BU. All the main peaks detected in batch 1 coincided with batch 2, demonstrating a good reproducibility of HRT composition.

### Cell culture and osteoclast formation

BMMs and primary osteoblasts were prepared from bone marrow cells (BMCs) in femur and calvarias of newborn mice as described previously [15]. To generate osteoclasts, BMMs (1 × 10^4^ cells/well in a 96-well plate) were cultured with M-CSF (60 ng/ml) and RANKL (150 ng/ml) for 4 days. BMCs (1 × 10^4^ cells/well) and primary osteoblasts (1 × 10^5^ cells/well) were cocultured for 6 days with 1,25(OH)_2_D_3_ (1 × 10^-8^ M). A CCK-8 was used to examine cell viability according to the manufacturer’s protocol. Cells were fixed and stained for TRAP assay. Total TRAP activity was measured at 410 nm after treatment with *p*-nitrophenyl phosphate. TRAP positive multinuclear cells (TRAP(+)MNCs) containing more than three nuclei and larger than 100 μm in diameter were counted. Data represented the mean ± SD of triplicate.

### Real-time quantitative PCR (QPCR)

To evaluate NF-κB- or NFATc1-regulated gene expression, cells (3 × 10^5^ cells/well in a 6-well plate) were pre-incubated with sample and M-CSF (60 ng/ml) for 2 h, and then stimulated with RANKL (150 ng/ml) for indicated times. Total RNA was isolated with an RNA-spin total RNA Extraction Kit according to the manufacturer’s protocol. cDNA was synthesized from 1 μg of total RNA in AccuPower RT-PreMix according to the manufacturer’s protocol. SYBR green-based QPCR amplification was performed using cDNA diluted to 1:3, 10 pmol of primers, and AccuPower GreenStar qPCR Master Mix in the Applied Biosystems 7500 Real-Time PCR System (Foster City, CA, USA). All reactions were run in triplicate, and data were analyzed using the 2^-ΔΔCT^ method. HPRT was used as the internal standard.

### Western blot analysis

BMMs were collected and lysed in RIPA buffer with protease-inhibitor and phosphatase-inhibitor cocktail tablet. Cell lysates were centrifugated at 10,000 × g for 15 min at 4°C. Protein samples were subjected to 10% polyacrylamide gel using a Mini Protean 3 Cell (Bio-Rad, Hercules, CA, USA) and then transferred onto a PVDF membrane. The membrane was first incubated in blocking buffer (10 mM Tris–HCl [pH 7.5], 150 mM NaCl, 0.1% Tween 20, and 5% non-fat dry milk), and then sequentially incubated with primary antibody (1 : 1000) and secondary antibody (1 : 2000) for 1 h at room temperature. Immunodetection was performed using SuperSignal West Femto Maximum Sensitivity Substrate. Chemiluminescent signals were detected with a LAS-4000 Luminescent Image Analyzer (Fuji Photo Film Co., Japan).

### Retrovirus preparation and infection

A DNA fragment from a constitutively active form of NFATc1 encoding HA-tagged Ca-NFATc1 (the XhoI/Klenow-EcoRI fragment) was subcloned into the pMX-puro vector (BamHI/Klenow-EcoRI) with puromycin as the selective marker (pMX-Ca-NFATc1). Preparation of retroviral stocks and viral infection were performed as described previously [15]. Retroviral infected BMMs were allowed to differentiate into osteoclasts for an additional 4–5 days in the presence of sample (10 μg/ml), M-CSF (60 ng/ml), and RANKL (150 ng/ml).

### Animal model of OVX rats

Sprague–Dawley female rats (10 weeks old) obtained from Samtako Bio Inc. (Seoul, Korea) were housed at 22 ± 1°C and 55 ± 10% humidity on a 12-h light/dark cycle with free access to food and water. Animal experiments were carried out in accordance with the National Institute of Health’s Guidelines for the Care and Use of Laboratory Animals. The experiments were approved by the Institutional Animal Care and Use Committee at the Korea Institute of Oriental Medicine. The rats were randomly divided into sham-operated (sham, *n* = 8) or surgically OVX (OVX, *n* = 24). One week after surgery, the OVX rats were randomly divided into three groups with eight rats each: (1) OVX: bilaterally OVX administered with saline; (2) HRT: bilaterally OVX administered with 0.3 g/kg of HRT; (3) fHRT: bilaterally OVX administered with 0.3 g/kg of fHRT. Oral administration of samples once a day began 1 week and finished 3 months after OVX surgery. Dosage for oral administration was based on maximum dosage for daily administration of herbal medicine in traditional herbal medicine clinic. The same amount of saline was orally administered to the sham and OVX groups.

### Micro-CT analysis

Computed tomographic images of the femur of each rats were acquired 3 month after OVX surgery, using the In-Vivo Micro-CT (SkyScan 1076, SkyScan N.V., Belgium) at a resolution of 18 μm, with the following parameters: 100 kV, 100 mA, 1770 ms. The beam-hardening errors were corrected to improve the quality of the micro-CT images by flat-field correction before scanning and beam-hardening correction during reconstruction. Three-dimensional models of the trabecular bone of femur were reconstructed using SkyScan CT Analyzer version 1.11 to evaluate the alteration of bone and the structural parameters.

### Statistical analysis

Statistical difference in TRAP activity and mRNA levels of genes were analyzed by Student’s *t*-test. Results of the animal experiment were analyzed using one-way analysis of variance followed by post-hoc Tukey HSD test for multiple comparisons. A *p* value less than 0.05 were considered significant. The statistical analysis was performed with Statistical Package for Social Sciences (SPSS10).

## Results

### HPLC analysis of HRT-BU and fHRT-BU

**Figure 1:**
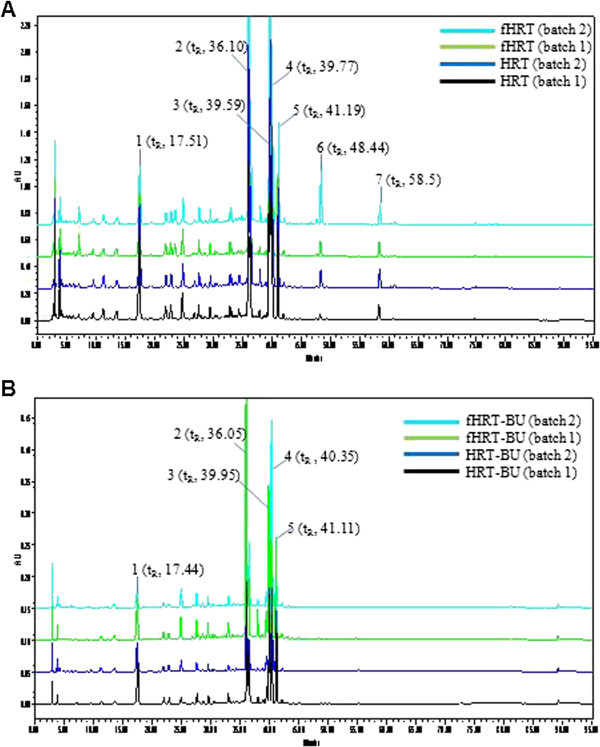
**The HPLC analysis of HRT and fHRT (A), HRT-BU and fHRT-BU (B) at 254 nm.** (1) geniposide, (2) baicalin, (3) palmatine, (4) berberine, (5) wogonoside, (6) baicalein, (7) wogonin.

To distinguish the difference of components between non-fermented and fermented HRT, we characterized the seven marker components in HRT by HPLC analysis (Figure 1). We found that geniposide (−28%) was decreased in fHRT compared to HRT, but baicalin (+249%) was increased in fHRT-BU compared to HRT-BU.

### HRT-BU and fHRT-BU inhibit osteoclastogenesis in BMMs and BMCs-osteoblast coculture

**Figure 2:**
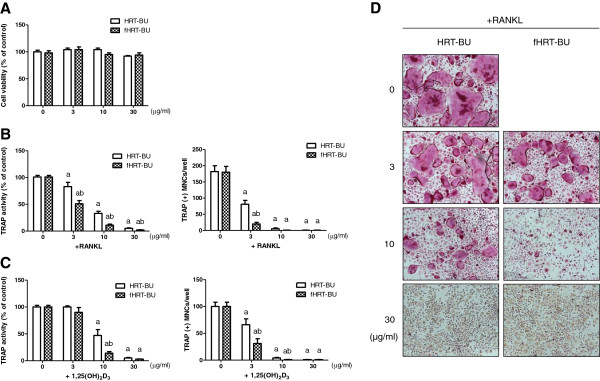
**Inhibitory effects of HRT-BU and fHRT-BU on osteoclast formation in BMMs and BMCs-osteoblast coculture.** (**A**) BMMs were incubated with M-CSF (60 ng/ml) and the indicated concentrations of HRT-BU or fHRT-BU for 3 days, and then cell viability of BMMs was measured by CCK-8 assay. (**B**) BMMs were incubated with M-CSF, RANKL (150 ng/ml), and the indicated concentrations of each sample for 4 days. (**C**) BMCs and primary osteoblasts were cocultured with the indicated concentrations of each sample and 1,25(OH)_2_D_3_ (1 × 10^-8^ M) for 6 days. The cells were stained for TRAP activity and the number of TRAP-positive multinuclear osteoclasts (TRAP(+)MNCs) was counted. (**D**) Representative microscopic pictures of RANKL-induced multinuclear osteoclasts were shown (100×). Data are mean ± SD (*n* = 3). ^a^*P* < 0.05, versus vehicle. ^b^*P* < 0.05, versus HRT-BU.

We evaluated the effect of HRT-BU and fHRT-BU on RANKL-induced osteoclastogenesis in BMMs. HRT-BU and fHRT-BU dose-dependently inhibited TRAP activity without cytotoxicity (Figure 2A and B). Especially, fHRT-BU exhibited a greater inhibitory effect on TRAP activity and the formation of TRAP-positive multinuclear cells than HRT-BU. In addition, 10 μg/ml concentrations of HRT-BU and fHRT-BU completely inhibited the formation of TRAP-positive multinuclear cells (Figure 2B and D). To confirm the inhibitory effect of HRT-BU and fHRT-BU on RANKL-induced osteoclastogenesis, we next examined the effect of HRT-BU and fHRT-BU on osteoclastogenesis in BMCs-osteoblast coculture condition. In this culture condition, fHRT-BU also showed a greater inhibitory effect than HRT-BU (Figure 2C). Although the inhibitory effect of HRT-BU at 3 μg/ml on MNC counting in BMM culture was greater than that of HRT-BU in coculture, there was no statistical significance. To emphasize the difference of inhibitory effect between HRT-BU and fHRT-BU, we subsequently used 10 μg/ml concentrations of fHRT and HRT in the following study.

### fHRT-BU inhibits RANKL-induced NFATc1 expression

**Figure 3:**
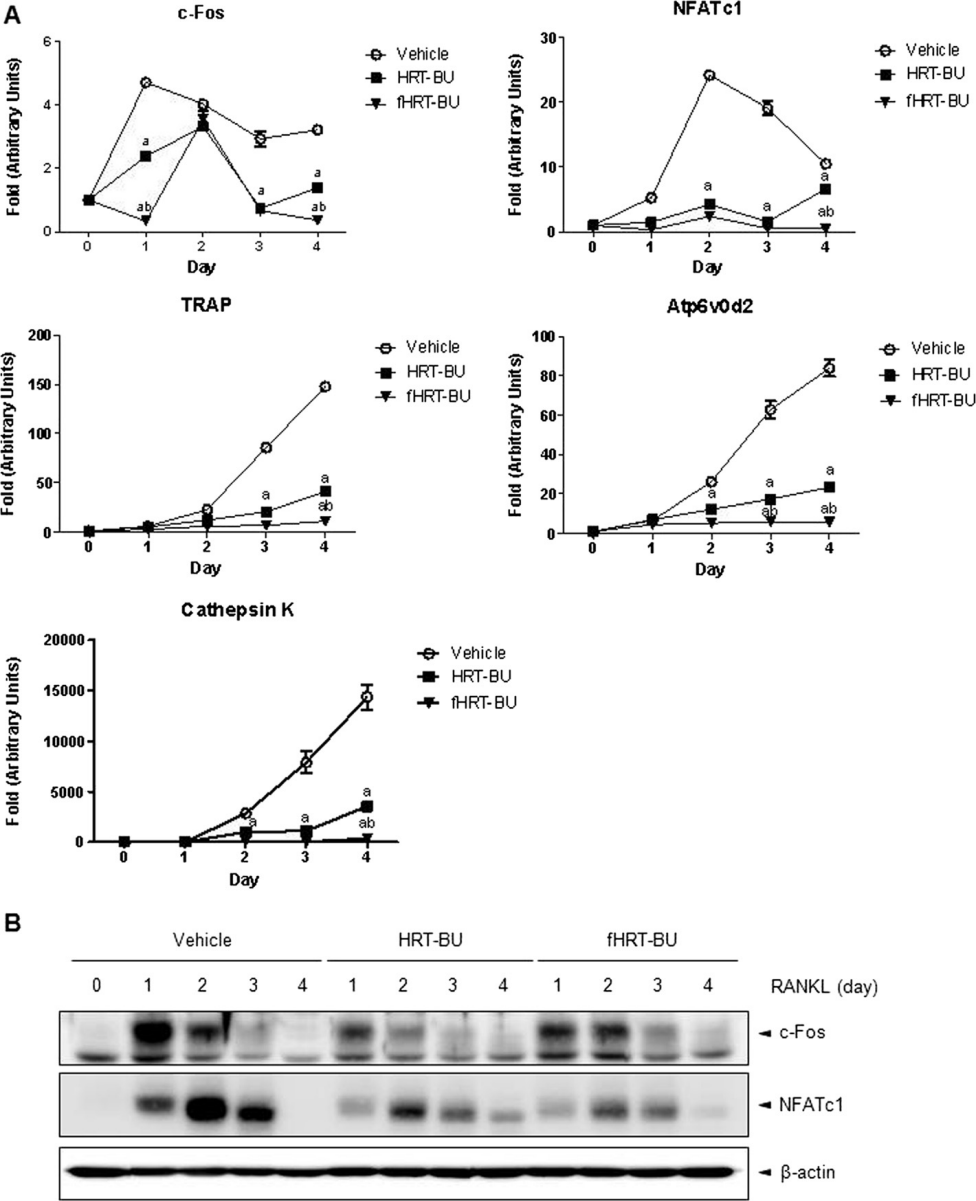
**Inhibitory effects of fHRT-BU on RANKL-induced NFATc1 expression in BMMs.** (**A**) BMMs were incubated with M-CSF (60 ng/ml), RANKL (150 ng/ml), and sample (10 μg/ml) for the indicated time points. mRNA expression of NFATc1, c-Fos, TRAP, ATPv0d2, and cathepsin K was analyzed by QPCR. (**B**) Whole cell lysates (30 μg) was analyzed by Western blot analysis with antibodies specific for NFATc1 and c-Fos. β–actin used as loading control. Data are mean ± SD (*n* = 3). ^a^*P* < 0.05, versus vehicle with RANKL, ^b^*P* < 0.05, versus HRT-BU with RANKL.

c-Fos and NFATc1 are key transcription factors for osteoclastogenesis. To determine the molecular mechanism regulated by HRT-BU and fHRT-BU on RANKL-induced osteoclastogenesis, we next explored mRNA expression of NFATc1 and c-Fos. HRT-BU significantly decreased mRNA levels of c-Fos about 2 folds less than vehicle at day 1 and NFATc1 about 6 folds less than vehicle at day 2, respectively (Figure 3A). Notably, fHRT-BU significantly decreased mRNA levels of c-Fos about 14 less than vehicle and NFATc1 about 10 fold less than vehicle at same day. We also investigated mRNA levels of NFATc1-regulated osteoclastogenesis related genes, including TRAP, ATP6v0d2, and cathepsin K. HRT-BU significantly decreased mRNA levels of osteoclastogenesis related genes about 4 fold less than vehicle at day 4. Furthermore, fHRT-BU significantly decreased mRNA levels of osteoclastogenesis related genes about 13 to 40 fold less than vehicle at day 4. To confirm these inhibitory effects of HRT-BU and fHRT-BU on c-Fos and NFATc1 expression, the protein expression of c-Fos and NFATc1 was determined by Western blot analysis. fHRT-BU decreased c-Fos expression, which was less than the inhibitory effect of HRT-BU on c-Fos expression (Figure 3B). However, when compared to vehicle and HRT-BU, fHRT-BU inhibited RANKL-induced NFATc1 expression at day 2, which is a time point that NFATc1 profoundly amplifies itself during osteoclastogenesis.

### fHRT-BU inhibits RANKL-induced MAP kinases activation

**Figure 4:**
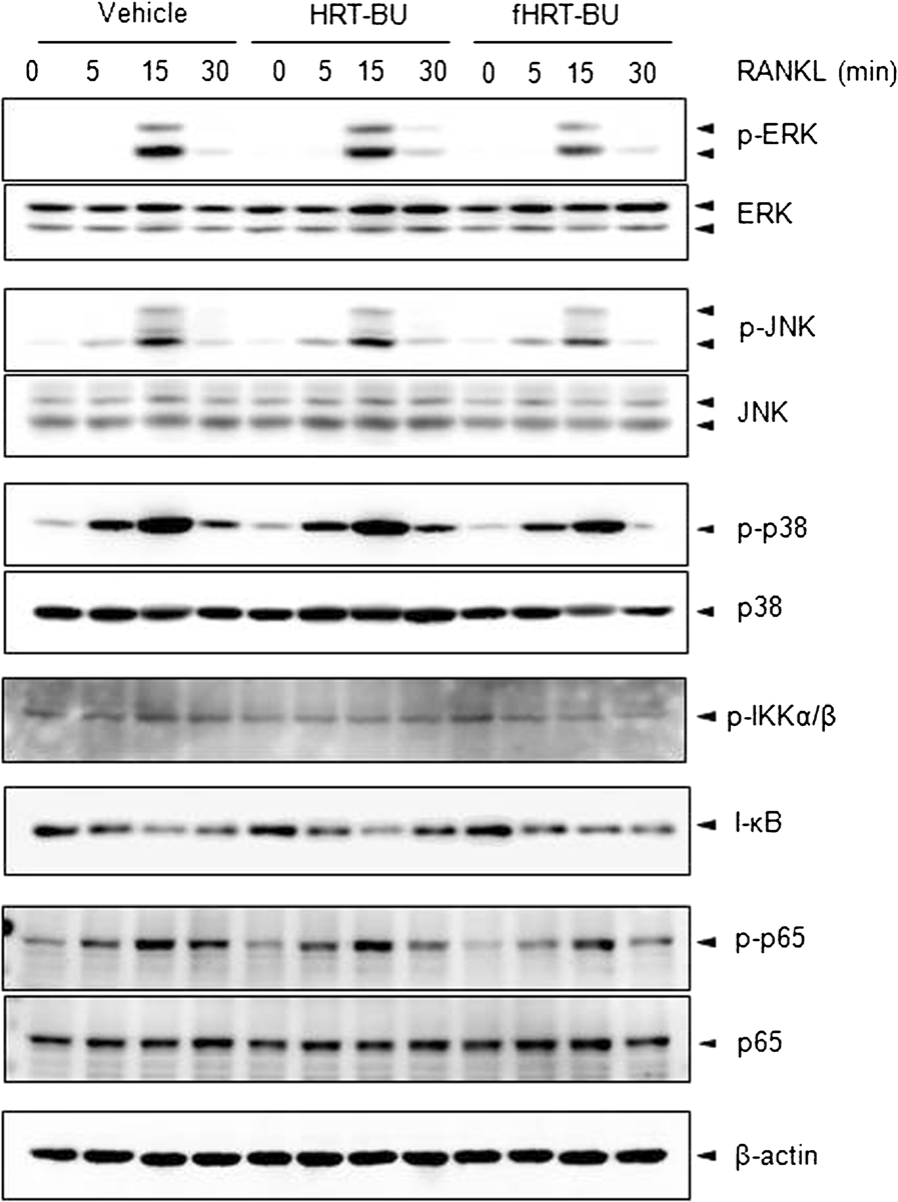
**Inhibitory effects of fHRT-BU on RANKL-induced ERK and JNK phosphorylation in BMMs.** BMMs were pre-treated with sample (10 μg/ml) for 2 h and then stimulated with RANKL (150 ng/ml) for the indicated time points. Whole cell lysates (10 μg) was analyzed by Western blot analysis with indicated antibodies. β–actin was used as loading control.

MAP kinases and NF-κB are RANKL-induced signaling pathways at early stage of osteoclastogenesis. To examine whether HRT-BU and fHRT-BU affect the activities of these pathways, BMMs were treated with HRT-BU or fHRT-BU and then were stimulated with RANKL for the indicated periods of time. HRT-BU did not affect the RANKL-induced activation of these signaling molecules (Figure 4). But, fHRT-BU partially decreased ERK and JNK activation.

### Ectopic expression of NFATc1 could not restores osteoclast formation suppressed by fHRT-BU

**Figure 5:**
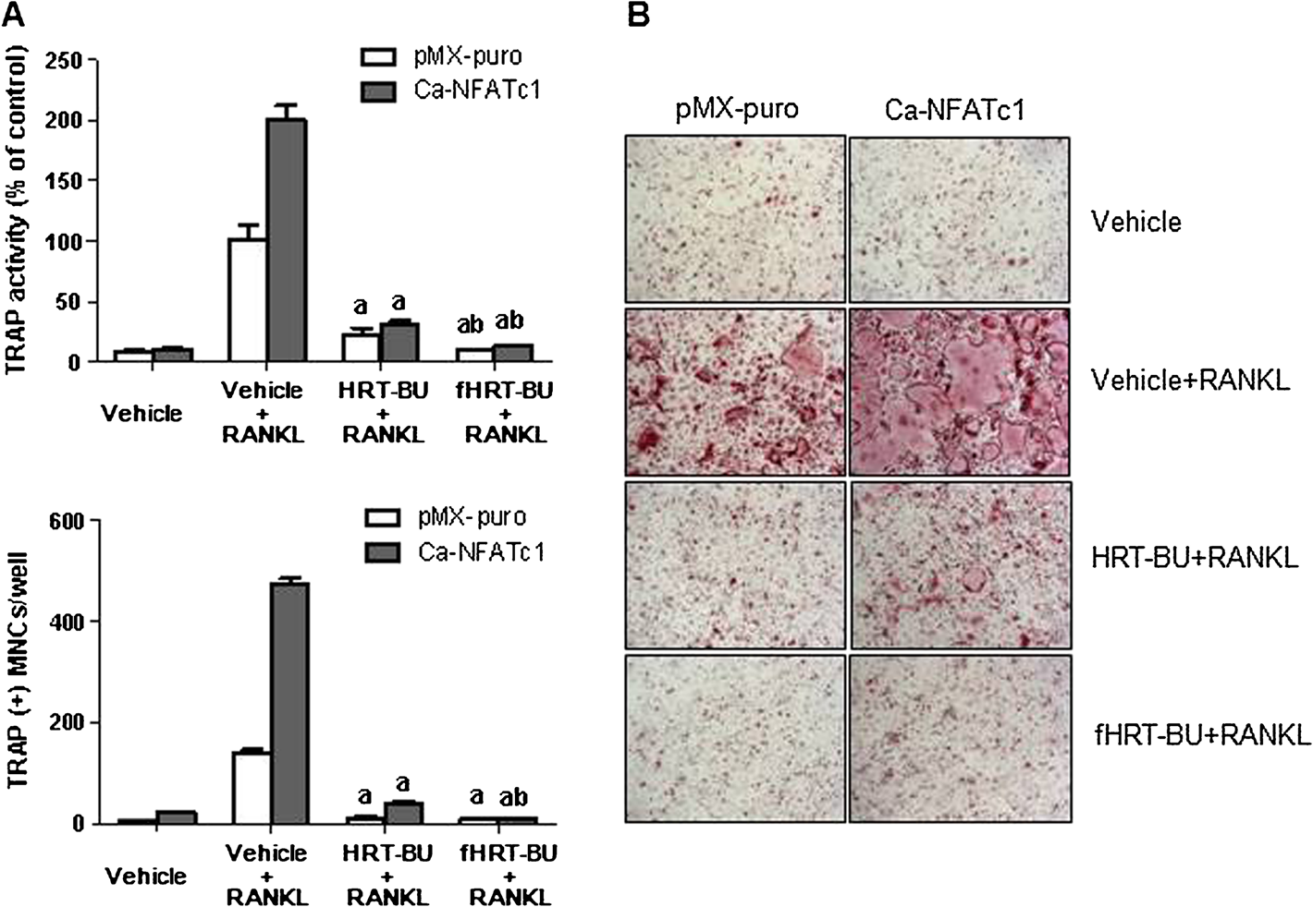
**Inhibitory effects of fHRT-BU on osteoclast formation induced by ectopic expression of NFATc1 in BMMs.** (**A**) BMMs were infected with pMX-puro (control) or retrovirus encoding HA-tagged Ca-NFATc1 (constitutively active form) and selected with puromycin (2 μg/ml) for 24 h. BMMs infected with retrovirus were incubated with M-CSF (60 ng/ml), RANKL (150 ng/ml), and sample (10 μg/ml) for 4 days. The cells were stained for TRAP activity and the number of TRAP-positive multinuclear osteoclasts (TRAP(+)MNCs) were counted. (**B**) Representative microscopic pictures of the cells (100×). Data are mean ± SD (*n* = 3). ^a^*P* < 0.05, versus vehicle with RANKL, ^b^*P* < 0.05, versus HRT-BU with RANKL.

NFATc1 is master transcription factor for osteoclastogenesis. Ectopic expression of NFATc1 induces efficient osteoclastogenesis in the absence of RANKL and restores osteoclastogenesis in the c-Fos knockout mouse [9]. Because HRT-BU and fHRT-BU significantly inhibited NFATc1 mRNA and protein expression, we explored whether overexpression of NFATc1 is sufficient to trigger osteoclastogenesis suppressed by fHRT-BU. We introduced a recombinant retroviral construct encoding a constitutively active form of NFATc1 (Ca-NFATc1) into osteoclast precursor cells. We found that ectopic expression of Ca-NFATc1 could not fully reduce the osteoclastogenesis inhibitory effect of HRT-BU and fHRT-BU (Figure 5). This result suggests that HRT-BU and fHRT-BU can act as an inhibitor of NFATc1 activity or its downstream signaling.

### fHRT prevents OVX-induced bone loss

**Figure 6:**
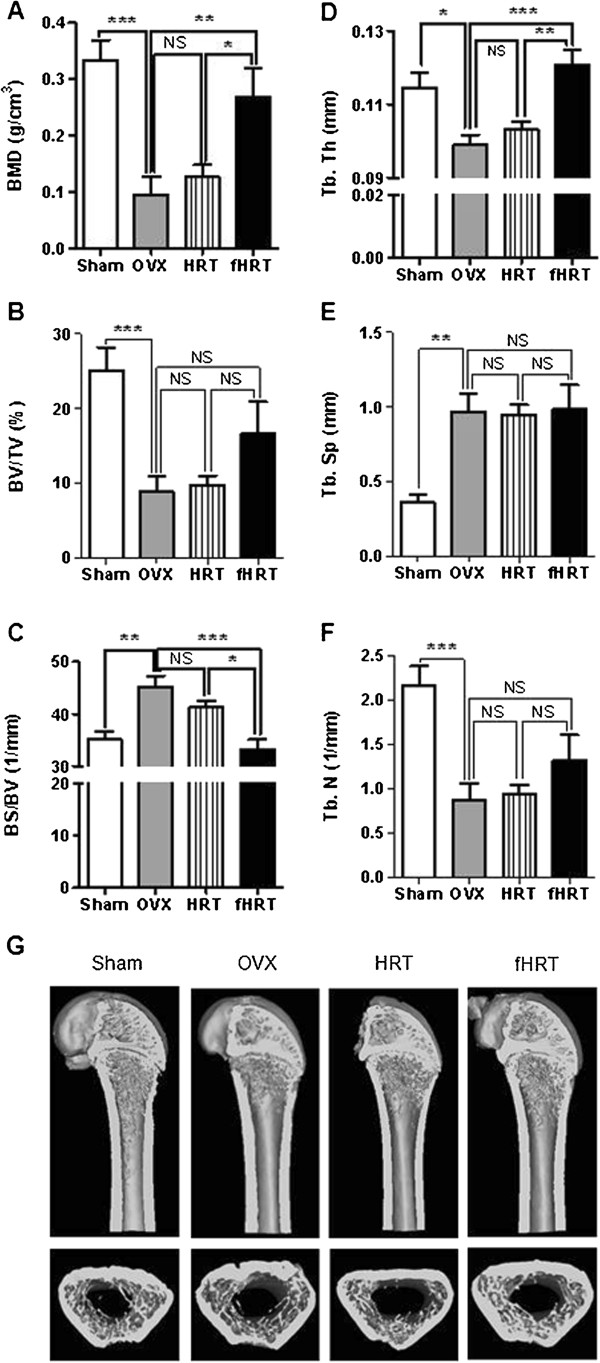
**Effect of fHRT on the bone parameters of OVX rats.** BMD and bone parameter of femur were analyzed by micro-CT after 12 weeks of HRT and fHRT administration in OVX rats. Graphs represented BMD (**A**), bone volume **(BV/TV, B)**, bone surface **(BS/BV, C)**, trabecular thickness **(Tb. Th, D)**, trabecular separation **(Tb. Sp, E)**, and trabecular number **(Tb. N, F)**. Data are mean ± SEM. *P < 0.05, **P < 0.01, ***P < 0.001. NS, not significant **(G)** Representative micro-CT images of femur of sham, OVX, HRT, and fHRT administrated groups.

We assessed the effect of HRT and fHRT on OVX-induced decrease of BMD and bone microstructure by micro-CT analysis. The OVX group significant decreased in 70% of BMD compared to sham group (Figure 6A). In addition, the OVX group exhibited significant change of bone parameters in BV/TV (−65%), BS/BV (+29%), Tb. Th (−14%), Tb. Sp (+169%), and Tb. N (−60%). The administration of fHRT (0.3 g/kg/day) to the OVX group significantly recovered BMD (+81%), BS/BV (−34%), and Tb.Th (19%) compared to OVX or HRT group (Figure 6A, C, and D). Although the administration of HRT to the OVX group slightly recovered BMD and BS/BV, there was no statistical significance.

## Discussion

In this study, we found that fHRT-BU significantly decreases RANKL-induced mRNA and protein expression of NFATc1. The molecular mechanisms underlying the inhibitory effect of fHRT on NFATc1 could be involved in 1) the transcriptional induction of NFATc1 expression, 2) the transcriptional activity of NFATc1 auto-amplification, or 3) the transcriptional activity of NFATc1 for its downstream signaling [8]. In this study we found that fHRT-BU greatly inhibited RANKL-induced NFATc1 expression than vehicle or HRT-BU at day 2, which is a time point that NFATc1 profoundly auto-amplifies NFATc1 expression itself [16]. We also found that fHRT-BU inhibits an ectopic and endogenous expression of NFATc1-induced osteoclastogenesis, which is similar level of fHRT inhibition on endogenous expression of NFATc1-induced osteoclastogenesis. Therefore, it might suggest that fHRT-BU acts as an inhibitor of NFATc1 transcriptional activity for its auto-amplification. In addition, we also found that fHRT-BU, but not HRT, partially affects ERK and JNK activation, which regulates RANKL-induced c-Fos expression through MEK1/2 or MKK7 pathway [17,18]. c-Fos is a component of AP-1 inducing NFATc1 expression in cooperation with other transcription factor, such as NF-κB [19]. We found that fHRT-BU did not regulate NF-κB activation. Thus, it suggests that fHRT might be involved in inhibiting MAP kinase-AP-1 pathway to regulate the transcriptional induction of NFATc1 on osteoclast differentiation [8,20,21].

Bacterial fermentation increases or generates the active components that have beneficial effect on bone. Our results showed that fermentation of HRT generates the different flavonoid profile compared to non-fermented HRT. 6-O-acylated isoflavone glycoside generated from soybeans fermentation with *Bacillus subtilis* or 1,4-dihydroxy-2-naphthoic acid, metabolic by-products of *Propionibacterium freudenreichii*, has a preventive effect on bone resorption or FK506-induced osteoporosis [22,23]. Inhibition of osteoclastic bone resorption and/or stimulation of osteoblastic bone formation maintain bone homeostasis by reducing bone turnover in bone disease [1]. Therefore, bacterial fermentation might generate the inhibitory components on osteoclastogenesis, which enhances the inhibitory effect of fHRT on OVX-induced bone loss and decreased bone parameter. In contrast to generation of the inhibitory molecules on osteoclasts by fermentation, fermentation also generates the anabolic molecules [13,24]. Since some flavonoids inhibiting osteoclastogenesis also have an anabolic activity on osteoblast differentiation, the stimulation of osteoblast differentiation might be other way of action mechanism of fHRT inhibiting OVX-induced bone loss. However, fHRT significantly inhibited osteoclastogenesis in coculture condition using BMMs and primary osteoblast, or in single culture condition using BMMs or RAW264.7 cells without osteoblasts (data not shown). Thus, it might suggest that fHRT directly acts on the differentiation of osteoclast precursor cells rather than the differentiation of osteoblasts.

Bacteria fermentation not only generates the bioactive components of flavonoid but also changes its structure. Bacterial fermentation de-glycosylates, sulfates, or methylates flavonoids, which influences the absorption rate and metabolism in the liver [25]. The structure change of flavonoid by bacteria fermentation increases the absorption rate and the amounts absorbed, which might elevate the bioactivity and bioavailability of active components [26,27] and might contribute to the beneficial effect on bone [28]. In this study, we found that fHRT shows a change of the marker component profile compared to HRT. We also found that HRT has no effect on bone loss but fHRT significantly inhibits OVX-induced bone loss. Therefore, these findings suggest that bacteria fermentation might affect the structure of bioactive components, the absorption rate, or the amounts absorbed in body which at least contributes to the inhibitory effect of fHRT on osteoclastogenesis or bone loss.

In this study, we found that HRT fermented by *Lactobacillus curvatus* changes the concentration of geniposide (−28%), berberine (+2.5%), and palmatine (+4%) when compared to HRT. However, HRT fermented by *Lactobacillus casei* slightly changed the concentration of these components with inhibiting osteoclastogenesis by inhibiting p38 and NF-κB p65 activation in our unpublished study (data not shown). Thus, it might suggest that different source of fermentation affects the pharmacological contents of herbal medicine, which is involved in the different mechanism of action on herbal medicine.

## Conclusions

We demonstrated that fHRT has an inhibitory activity on RANKL-induced osteoclastogenesis by suppressing NFATc1 expression, which might result in an improvement of BMD and bone parameter in OVX rats. Our results suggest that bacteria fermentation enhances the inhibitory effect of HRT on postmenopausal osteoporosis. Further studies are required to identify the active components in fHRT.

## Abbreviations

AP-1: Activation protein-1; ATPv0d2: The d2 isoform of vacuolar ATPase V0 domain; BMCs: Bone marrow cells; BMD: Bone mineral density; BMM: Bone marrow-derived macrophage; ERK: Extracellular signal-regulated kinase; JNK: c-Jun N-terminal kinase; HRT: Hwangryun-haedok-tang; fHRT: Fermented HRT; MAP: Mitogen-activated protein; M-CSF: Macrophage colony-stimulating factor; NFATc1: Nuclear factor of activated T cells cytoplasmic 1; NF-κB: Nuclear factor-κB; OVX: Ovariectomy; RANKL: Receptor activator for the nuclear factor-κB ligand; TRAP: Tartrate-resistant acid phosphatase

## Competing interests

The authors declared that they have no competing interests.

## Authors’ contributions

KSS and TSK carried out the experiments and analyzed the data. HIH and JYM designed the study and wrote the manuscript. KJL carried out HPLC analysis. CWC, HSK, and DHS carried out and analyzed animal experiments. All authors read and approved the final manuscript.

## Pre-publication history

The pre-publication history for this paper can be accessed here:

http://www.biomedcentral.com/1472-6882/13/106/prepub
